# Prolactinomas in the transition from adolescence to young adulthood: A multicentre, retrospective study from the TALENT group

**DOI:** 10.1111/jne.70275

**Published:** 2026-09-24

**Authors:** Dario De Alcubierre, Claudia Giavoli, Tiziana Feola, Lorenzo Cerroni, Ludovica Vincenzi, Eriselda Profka, Aurora Pedroli, Giulia Puliani, Rosa Pirchio, Marta Tenuta, Renata S. Auriemma, Maria Luisa Appetecchia, Rosario Pivonello, Giovanna Mantovani, Andrea M. Isidori, Marie‐Lise Jaffrain‐Rea, Ashley Grossman, Emilia Sbardella

**Affiliations:** ^1^ Department of Experimental Medicine Sapienza University Rome Italy; ^2^ Neuroendocrinology IRCCS Neuromed Institute Pozzilli Italy; ^3^ Endocrinology Unit Fondazione IRCCS Ca' Granda Ospedale Maggiore Policlinico Milan Italy; ^4^ Department of Clinical Sciences and Community Health, Department of Excellence 2023‐2027 University of Milan Milan Italy; ^5^ Oncological Endocrinology Unit IRCCS Regina Elena National Cancer Institute Rome Italy; ^6^ Dipartimento Di Medicina Clinica E Chirurgia, Sezione Di Endocrinologia, Diabetologia, Andrologia e Nutrizione Università Federico II Di Napoli Naples Italy; ^7^ Rare Endocrine Diseases Endo‐ERN Unit Azienda Ospedaliera Universitaria “Federico II” Naples Italy; ^8^ Staff of Unesco for Health Education and Sustainable Development University “Federico II” Naples Italy; ^9^ Centre for Rare Diseases (Endo‐ERN accredited) Policlinico Umberto I Rome Italy; ^10^ Department of Biotechnological and Applied Clinical Sciences University of L'Aquila L'Aquila Italy; ^11^ Green Templeton College University of Oxford Oxford UK; ^12^ Centre for Endocrinology, Barts and the London School of Medicine Queen Mary University of London London UK

**Keywords:** adolescents, cabergoline, dopamine agonist resistance, prolactinoma, transition age

## Abstract

Prolactinomas in the transition age present unique challenges; treatment strategies and long‐term outcomes in this population remain incompletely characterised. This is a multicentre, retrospective study of 110 consecutive patients (33 males) with prolactinomas, aged 15–25 years, across five Italian referral centres (2010–2025). Clinical, hormonal, radiological, and treatment‐related parameters were assessed at diagnosis and at follow‐up. At diagnosis, the median age was 20 years; median prolactin 177 ng/mL [49–6646]; 55% of patients had micro‐tumours. Male patients presented with larger tumours, higher prolactin levels, and greater invasiveness (*p* < .001 for all comparisons). Cabergoline was first‐line therapy in 95%, achieving prolactin normalisation in 75% and significant tumour shrinkage (≥50% volume and/or diameter reduction) in 66% of patients. Partial resistance (biochemical and/or radiological) occurred in 40% of patients. Treatment discontinuation occurred in 48% of cases; 61% of patients discontinuing cabergoline after prolonged normalisation experienced recurrence, which was predicted by younger age at diagnosis (OR 0.67/year, *p* = .040) and shorter treatment duration (OR 0.95/month, *p* = .050), the latter showed 93% sensitivity for recurrence at 45.5 months. Adverse events occurred in 12%, leading to treatment discontinuation in 6%. Surgery was required in 17%, achieving long‐term remission in 31%, while 21% of surgical cases required radiotherapy. At last follow‐up (median 72 months), 73% remained on cabergoline while 25% achieved complete radiological resolution. Transition‐age prolactinomas demonstrate high cabergoline resistance rates and frequent recurrence after discontinuation. Treatment duration ≥45.5 months before withdrawal may reduce recurrence risk. Surgical remission is achievable, especially in non‐invasive tumours, although multimodal therapy is often required for disease control.

## 1 BACKGROUND

Prolactinomas are the most common subtype of pituitary neuroendocrine tumours (PitNETs), accounting for over half of such tumours in the general population.^1^ However, these tumours are much rarer in children and adolescents, with an estimated prevalence of approximately 1 in 10,000 (or 100 per million),^2^ considerably lower than in adults,^3^, ^4^, ^5^ with a median age at diagnosis of 16 years (range 13–19) and marked female predominance.^6^, ^7^, ^8^ Clinical presentation demonstrates notable sex differences: females typically present with hyperprolactinaemia‐related symptoms, such as menstrual disturbances and galactorrhoea, whereas males more frequently exhibit mass‐related symptoms such as headache, visual field defects, and hypopituitarism, leading to reduced height velocity and delayed puberty.^3^, ^4^ Importantly, macro‐tumours (tumours ≥10 mm) are disproportionately more prevalent in the adolescent population, with an even higher proportion observed in males,^4^, ^6^ who also present with higher prolactin (PRL) levels.^3^

The pathophysiological mechanisms underlying the more aggressive phenotype observed in adolescent prolactinomas remain incompletely understood. Germline mutations in genes associated with pituitary tumourigenesis, such as the aryl hydrocarbon receptor‐interacting protein (*AIP*) gene or multiple endocrine neoplasia type 1 (MEN1), have been identified in approximately 11%–14% of young patients with macroprolactinomas^9^, ^10^ and have been suggested to influence tumour behaviour and response to dopamine agonists (DA).^9^, ^11^ DAs, particularly cabergoline (CAB), remain the first‐line treatment for prolactinomas in all age groups.^3^, ^12^ Most paediatric and adolescent prolactinomas show a favourable biochemical and radiological response to DAs, although resistance is reported more frequently than in adults, particularly in males and/or with large or invasive tumours.^3^, ^13^, ^14^, ^15^ Surgical intervention is required in 28%–45% of adolescent patients, either as primary therapy (i.e., in the presence of visual compromise, for large invasive tumours, or even as a curative option in selected cases at experienced pituitary centres, particularly for well‐circumscribed lesions),^12^ or as a secondary treatment following DA resistance or intolerance.^3^, ^16^ Reported long‐term surgical remission rates remain variable and strongly affected by tumour invasiveness.^3^

The transition from paediatric to adult endocrinology care represents a particularly vulnerable phase for prolactinoma patients, coinciding with critical physiological changes, including completion of pubertal development, achievement of peak bone mass, and establishment of reproductive function—all processes potentially compromised by hyperprolactinaemia and its associated hypogonadism.^17^ Furthermore, adherence to long‐term medical therapy may be challenged by psychosocial factors characteristic of this age group. Recent international consensus guidelines highlight the need for age‐adapted management of prolactinomas in this population, emphasising structured transition care, selective genetic testing, and comprehensive long‐term follow‐up.^14^, ^15^ They also note the limited evidence for standardised dopamine agonist withdrawal criteria, supporting an individualised and cautious approach, particularly in higher‐risk patients.^14^, ^15^ Despite increasing recognition of paediatric prolactinomas, knowledge gaps in patients diagnosed during the transition age (15–25 years) still persist, particularly regarding optimal duration of DA therapy, criteria for treatment withdrawal, long‐term metabolic consequences, and strategies for managing treatment‐resistant disease.^12^, ^13^ This study aims to synthesise the clinical experience of a large Italian population of adolescent prolactinomas during the transition period, describing onset presentation and evolution, therapeutic management, and long‐term outcomes.

## 2 METHODS

### 2.1 Patients and study design

This real‐life, longitudinal, retrospective observational study included consecutive patients diagnosed with prolactinomas between 15 and 25 years at five Italian referral centres for pituitary disorders (IRCCS Neuromed Institute in Pozzilli, Fondazione IRCCS Ca′ Granda Ospedale Maggiore Policlinico in Milan, Sapienza University in Rome, Federico II University in Naples, IRCCS Regina Elena National Cancer Institute in Rome) between January 2010 and December 2025. Exclusion criteria included age at diagnosis <15 or >25 years and the unavailability of follow‐up assessments after initial diagnosis. This study was approved by the Local Ethics Committee of Sapienza University in Rome (coordinating centre, reference number 6525) and was conducted in accordance with the Declaration of Helsinki (1964) and its subsequent amendments. All enrolled patients provided their written informed consent to participate in the study.

### 2.2 Data collection

Clinical, hormonal, and radiological parameters were collected through a review of medical records and electronic databases at diagnosis and up to the last available follow‐up. PRL levels were measured in each centre using commercially available assays, and assay‐specific reference ranges were used to define PRL normalisation. Patients with biochemical evidence of GH/IGF‐1 hypersecretion were excluded. Hypopituitarism was defined according to current endocrine guidelines.^18^ Longitudinal, sagittal, and coronal non‐enhanced and/or enhanced T1‐weighted images, as well as coronal T2‐weighted images, were retrieved for all patients and evaluated by an endocrinologist with expertise in neuroradiology at each centre (Dario De Alcubierre, Claudia Giavoli, Giulia Puliani, Rosa Pirchio). Based on maximum tumour diameter, all prolactinomas were classified as microadenomas (<10 mm), macroadenomas (10–40 mm), or giant adenomas (>40 mm). Local invasiveness and suprasellar extension were also evaluated according to the Knosp classification for parasellar invasion and the Hardy–Wilson grading system for suprasellar extension.^19^ In patients undergoing pituitary surgery, pathological reports were obtained and immunohistochemical data on hormone expression, transcription factors and proliferation indices were collected. Whenever available, the prevalence of germline mutations in genes commonly associated with pituitary tumour susceptibility (*AIP*, *MEN1*, *CDKN1B*) was also recorded using targeted sequencing.

### 2.3 Treatment outcomes

Treatment history was collected for each patient. According to guidelines,^12^, ^20^, ^21^ and based on clinical picture and personal choice, patients were directed to either first‐line medical therapy with CAB or pituitary surgery. Significant tumour shrinkage was defined by a decrease of at least 50% in tumour volume and/or maximum diameter. Radiological follow‐up data was available for 103/110 patients. Resistance to CAB was defined as the failure to normalise PRL levels with a continuous dose of 2 mg/week and up to the maximum tolerable dose (4.5 mg/week) for at least 6 months, or as the failure to achieve significant tumour shrinkage on the maximally tolerated doses of DA, according to current criteria.^12^, ^20^ Partial resistance was defined by failure in a single domain: partial biochemical resistance as the failure to normalise PRL despite adequate radiological response (decrease of at least 50% in tumour volume and/or maximum diameter), and partial radiological resistance as the failure to achieve significant shrinkage despite PRL normalisation; combined resistance was defined by the coexistence of both criteria in the same patient. Cumulative CAB exposure was calculated at 6, 12, and 60 months of treatment and at the last available follow‐up, by multiplying the weekly dose by the number of treatment weeks, considering any dose adjustments or treatment interruptions.

### 2.4 Statistical analysis

The distribution of continuous variables was assessed using the Shapiro–Wilk test. Categorical variables were expressed as frequencies and percentages, whereas continuous variables were reported as the mean ± standard deviation (SD) or the median [5th–95th percentile], depending on data distribution. Subgroup comparisons for categorical variables (e.g., resistance rates by tumour size, invasiveness, extension patterns) were performed using Χ‐square tests or Fisher's exact tests, as appropriate. Continuous variables between groups were compared using Student's *t* or Mann–Whitney *U* tests, based on data distribution. For comparisons of continuous variables measured at two different timepoints within the same individuals, paired‐samples *t*‐tests or Wilcoxon signed‐rank tests were used, as appropriate. Categorical variables were compared using McNemar's test for paired proportions. Univariate logistic regression analyses were first performed to evaluate the association between clinically plausible candidate predictors of CAB resistance and recurrence following its discontinuation. The assumption of linearity between continuous variables and the logit of the outcome was assessed prior to regression analyses. Odds ratios (ORs) with 95% confidence intervals (CIs) and associated *p*‐values were reported. Variables showing the strongest associations in univariate analyses were subsequently included in a multivariable logistic regression model to estimate adjusted odds ratios. Time‐to‐event analyses for PRL normalisation and tumour shrinkage were performed using Kaplan–Meier methods, with log‐rank (Mantel–Cox) tests for group comparisons. Receiver operating characteristic (ROC) curve analysis was used to identify optimal cut‐off values for treatment duration predicting recurrence. Effect sizes were calculated as standardised β coefficients for regression analyses. A two‐sided *p*‐value <.05 was considered statistically significant. *p*‐Values between .05 and .10 were interpreted as a statistical trend. All analyses were conducted using SPSS version 29 (IBM Corp., Armonk, NY) and GraphPad Prism version 10.0 (GraphPad Software, La Jolla, CA).

## 3 RESULTS

### 3.1 Baseline characteristics

One‐hundred ten patients (33 males, 77 females) with prolactinomas diagnosed during the transition age, median age at diagnosis 20 years (15–25 years), were included in the study. Twenty‐eight patients (25.4%) were diagnosed before 18 years of age. Clinical, hormonal, and radiological characteristics at diagnosis are detailed in Table 1. Overall, 50 patients presented with macroadenomas (including five giant tumours), with a median PRL in the overall cohort of 177 ng/mL (49–6646). Twenty‐two patients (20%) exhibited invasive tumours. Genetic testing was performed in 32 cases (29%; 14 males, median age 20 years, 20 with macro‐tumours and 12 with micro‐tumours, 11 invasive); in no case did germline analysis identify pathogenic variants.

**TABLE 1:** Characteristics of the study cohort at diagnosis.

	All (*n* = 110)	Males (*n* = 33)	Females (*n* = 77)	*p*‐Value
Age, years	20 [15–25]	21 [15–25]	20 [15–25]	.839
PRL, ng/mL	177 [49–6646]	472 [38–12,349]	150 [50–878]	**<.001**
Micro/macro/giant tumours	60/45/5	9/19/5	51/26/0	**<.001**
Maximum diameter, mm	9.0 [3–38]	21.0 [4–46]	8.0 [3–19]	**<.001**
Tumour volume, mm^3^	266 [19–13,478]	3194 [35–27,492]	204 [14–2547]	**<.001**
Cystic lesion, *n* (%)	12 (10.9)	2 (6.0)	10 (12.9)	.286
Suprasellar extension, *n* (%)	26 (23.6)	17 (51.5)	9 (11.6)	**<.001**
Parasellar extension, *n* (%)	16 (14.5)	14 (42.4)	2 (2.5)	**<.001**
Infrasellar extension, *n* (%)	10 (9.1)	9 (27.2)	1 (1.3)	**<.001**
Incidental finding, *n* (%)	4 (3.6)	2 (6.0)	2 (2.5)	.374
Headache, *n* (%)	41 (37.2)	18 (54.5)	23 (29.8)	.**014**
Visual impairment, *n* (%)	14 (12.7)	12 (36.3)	2 (2.5)	**<.001**
Menstrual abnormalities, *n* (%)	66 (60)	‐	66 (85.7)	‐
Amenorrhoea, *n* (%)	41 (37.2)	‐	41 (53.2)	‐
Oligomenorrhoea, *n* (%)	25 (22.7)	‐	25 (32.4)	‐
Galactorrhoea, *n* (%)	47 (42.7)	6 (18.1)	41 (53.2)	.**001**
Sexual dysfunction, *n* (%)	18 (16.3)	16 (48.4)	2 (2.5)	**<.001**
Gynaecomastia, *n* (%)	16 (14.5)	16 (48.4)	‐	‐
Delayed puberty, *n* (%)	7 (6.3)	5 (15.1)	2 (2.5)	.**013**
BMI, kg/m^2^	23.7 [18.3–39.5]	25.9 [19.4–43.8]	22.5 [18.1–33.0]	**<.001**
Obesity, *n* (%)	13 (11.8)	7 (21.2)	6 (7.8)	.**046**
Hypertension, *n* (%)	1 (0.9)	1 (3.0)	0 (0)	.125
Type 2 diabetes mellitus, *n* (%)	0 (0)	0 (0)	0 (0)	‐
Dyslipidaemia, *n* (%)	2 (1.8)	0 (0)	2 (2.5)	.350
TSH deficiency, *n* (%)	8 (7.2)	3 (9.0)	5 (6.5)	.631
ACTH deficiency, *n* (%)	9 (8.1)	5 (15.1)	4 (5.2)	.081
FSH/LH deficiency, *n* (%)	31 (28.1)	20 (60.6)	11 (14.3)	**<.001**
GH deficiency, *n* (%)	3 (2.7)	1 (3.0)	2 (2.5)	.898
Follow‐up duration, months	72 [15–217]	71 [15–217]	84 [20–197]	.358

*Note*: Clinical, hormonal, and radiological features of 110 patients with prolactinomas diagnosed during transition age, stratified by sex. Categorical variables are presented as number (%). Continuous variables are presented as median [5th–95th percentile]. Bold values indicate statistically significant differences (*p* < 0.05).

Abbreviations: ACTH, adrenocorticotropic hormone; BMI, body mass index; FSH, follicle‐stimulating hormone; GH, growth hormone; LH, luteinizing hormone; PRL, prolactin; TSH, thyroid‐stimulating hormone.

As expected, marked sex‐related differences were observed: males exhibited higher PRL levels, larger maximum tumour diameter and volume, and a predominance of macro‐tumours compared to females (*p* < .001 for all); notably, giant prolactinomas (*n* = 5) occurred exclusively in males. Correspondingly, males exhibited higher rates of suprasellar, parasellar, and infrasellar extension (*p* < .001 for all). Galactorrhoea was more prevalent in females (*p* = .001), while males more frequently presented with sexual dysfunction (*p* < .001), headache (*p* = .014), and visual impairment (*p* < .001). Hypogonadotrophic hypogonadism was significantly more common in males (*p* < .001); however, no patient required priming for puberty induction. In females, amenorrhoea was secondary in 38/41 cases and primary in 3. Males exhibited a higher BMI (*p* < .001) and prevalence of obesity (*p* = .046).

### 3.2 Cabergoline treatment

**FIGURE 1:**
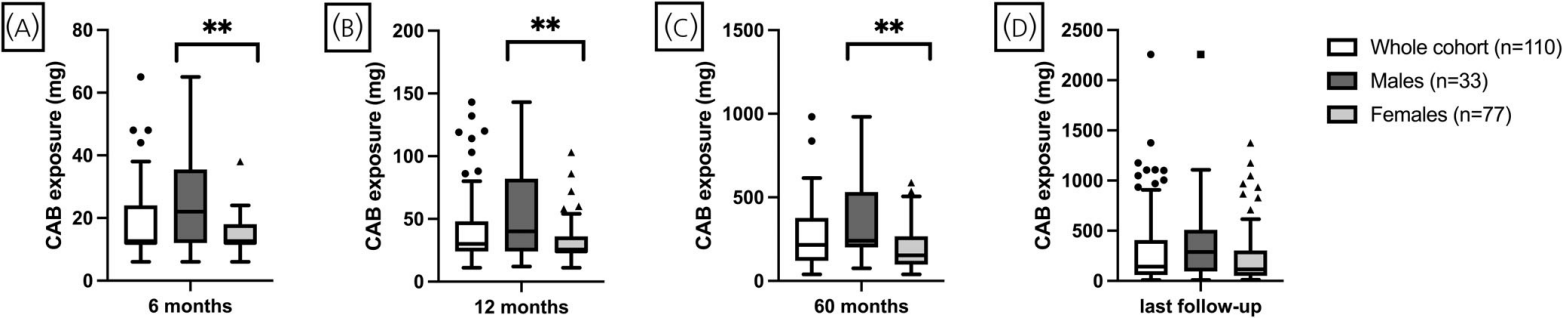
Cumulative cabergoline exposure over time, in the whole cohort and after stratification by sex. Box‐and‐whisker plots show cumulative cabergoline exposure at (A) 6 months, (B) 12 months, (C) 60 months, and (D) last available follow‐up in the whole cohort (white boxes), males (dark grey boxes) and females (light grey boxes). Boxes indicate the median and interquartile range, whiskers the Tukey range, and symbols represent individual patients. CAB, cabergoline.

Treatment‐related variables are detailed in Table 2. CAB was first‐line therapy in 95% of patients (all females and 85% of males, *p* = .002). The median starting dose was 0.5 mg/week [0.25–1.0], with a median treatment duration of 44 months [3–166]. The maximal CAB dose during treatment was 1.0 mg/week [0.25–3.5]; cumulative CAB exposure was higher in males at 6 (*p* = .003, Figure 1A), 12 (*p* = .005, Figure 1B), and 60 months (*p* = .007, Figure 1C), although not at the last available follow‐up (*p* = .084, Figure 1D), which may be explained by the heterogeneity of follow‐up duration, with patients still under dose titration.

**TABLE 2:** Characteristics of pharmacological treatment and response in the TALENT cohort of transition age‐onset prolactinomas.

	All (*n* = 110)	Males (*n* = 33)	Females (*n* = 77)	*p*‐Value
CAB first‐line therapy, *n* (%)	105 (95.4)	28 (84.8)	77 (100)	.**002**
CAB starting dose, mg/week	0.5 [0.25–1]	0.5 [0.25–2.0]	0.5 [0.25–1.0]	.224
First‐line CAB duration, months	44 [3–166]	58 [3–166]	39 [3–199]	.359
Maximal CAB dose during treatment, mg/week	1.0 [0.25–3.5]	1.0 [0.25–3.75]	0.75 [0.25–3.5]	.062
Cumulative CAB dose (6 months)	12.0 [6–38]	22 [8–58]	12 [6–24]	.**003**
Cumulative CAB dose (12 months)	30.0 [12–114]	40 [16–139]	24 [12–65]	.**005**
Cumulative CAB dose (60 months)	211.0 [50–715]	240 [75–851]	153 [41–475]	.**007**
Time to normalise PRL, months^a^	6.0 [1–41]	4.0 [1–57]	6.0 [2–41]	.595
Max CAB dose at PRL normalization, mg/week	0.5 [0.25–2.5]	1.0 [0.25–3]	0.5 [0.25–2.0]	.298
Cumulative CAB dose at PRL normalization, mg	12.0 [2–212]	9.0 [1–231]	14.0 [3–180]	.448
Significant tumour volume shrinkage during CAB, *n* (%)	68/103 (66.0)	17/29 (58.6)	51/74 (68.9)	.321
Significant diameter shrinkage during CAB, *n* (%)	52/103 (50.4)	14/29 (48.2)	38/74 (51.3)	.779
Max tumour volume shrinkage during CAB, %	81.9 [0–100]	82.2 [0–100]	81.2 [0–100]	.690
Max tumour diameter shrinkage during CAB, %	47.2 [0–100]	43.3 [0–100]	50.0 [0–100]	.819
Time to achieve tumour shrinkage, months^a^	24.0 [4–105]	36.0 [3–66]	24.0 [6–108]	.831
Max CAB dose at tumour shrinkage, mg/week	0.75 [0.25–3]	1.5 [0.25–3]	0.75 [0.12–2.5]	.**006**
Cumulative CAB dose at tumour shrinkage, mg	80 [12–445]	111 [15–514]	80 [12–339]	.122
Resistance to CAB, *n* (%)	44 (40.0)	16 (48.5)	28 (36.3)	.234
Biochemical resistance, *n* (%)	27 (24.5)	11 (33.3)	16 (20.7)	.134
Radiological resistance, *n* (%)	37 (33.6)	14 (42.4)	23 (29.8)	.129
Both, *n* (%)	19 (17.2)	9 (27.2)	10 (12.9)	.058

*Note*: Treatment‐related variables in the TALENT cohort of prolactinomas diagnosed during transition age, stratified by sex. Categorical variables are presented as number (%). Continuous variables are presented as median [5th–95th percentile]. Bold values indicate statistically significant differences (*p* < 0.05).

Abbreviations: CAB, cabergoline; PRL, prolactin.

^a^ Time‐to‐event variables in this table represent observed medians among patients achieving the outcome, whereas Kaplan–Meier analyses (Figure 2) estimate time to event accounting for censored observations.

**FIGURE 2:**
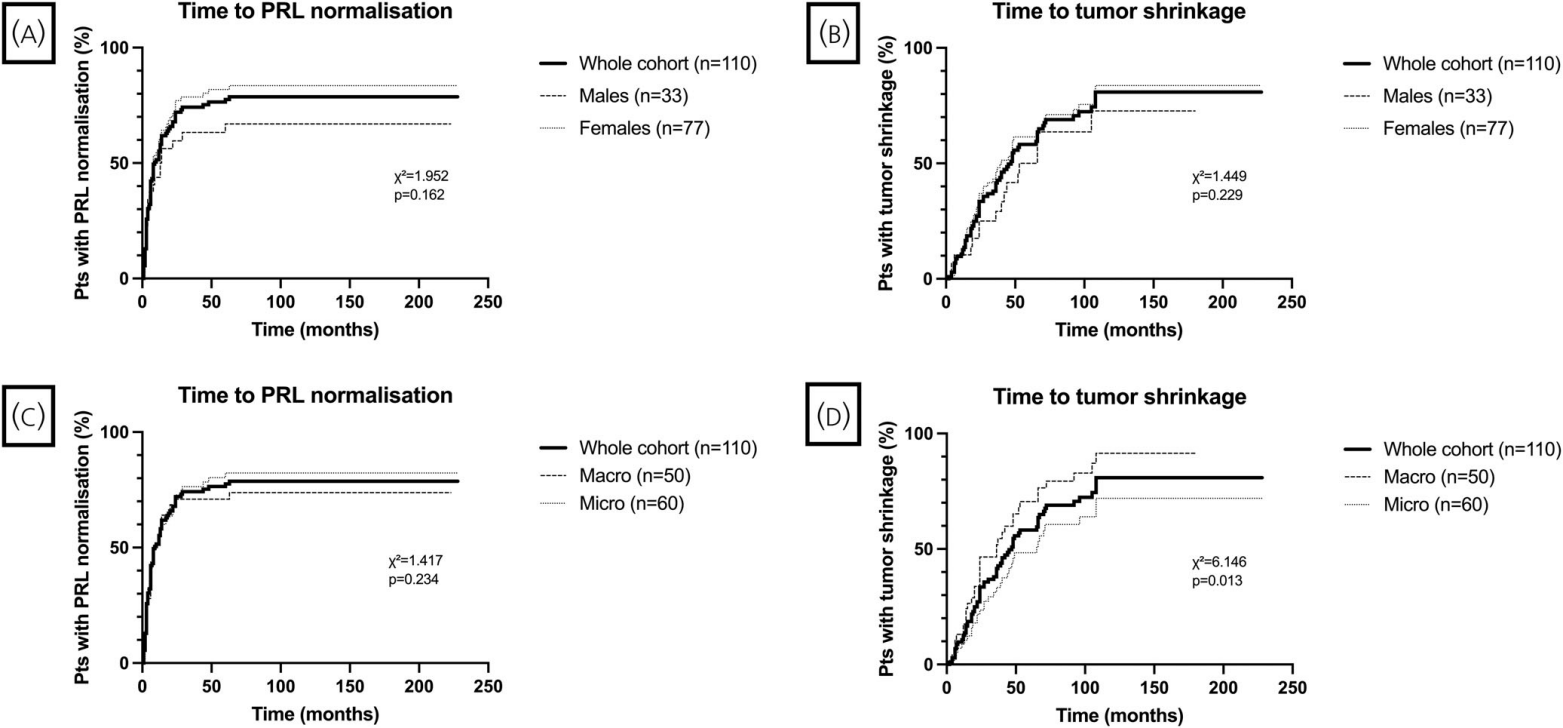
Cumulative incidence of PRL normalisation and tumour shrinkage, in the whole cohort and after stratification for sex and tumour size. (A, B) Kaplan–Meier curves showing the cumulative probability of (A) PRL normalisation and (B) significant tumour shrinkage over time in the whole cohort (bold line), females (dotted line), and males (dashed line). (C, D) Kaplan–Meier curves showing the cumulative probability of (C) PRL normalisation and (D) significant tumour shrinkage over time in the whole cohort (bold line), micro‐tumours (dotted line), and macro‐tumours (dashed line). Time is expressed in months. Comparisons between curves were performed using the log‐rank (Mantel–Cox) test.

PRL normalisation occurred in 75% of patients (83/110) at an observed median time of 6.0 months [1–41] with a maximal dose of 0.5 mg/week [0.25–2.5] and cumulative dose of 12.0 mg [2–212], and no significant difference between sexes (Table 2 *p* = .595, log‐rank test *p* = .227). Median volume shrinkage was 82% [0–100], comparable between sexes (*p* = .690), with males requiring higher CAB doses to achieve shrinkage (*p* = .006). Significant tumour volume shrinkage (≥50% reduction from baseline) was achieved in 66% (68/103) of patients after an observed median time of 24 months [4–105] without significant sex differences (*p* = .321). Sex stratification revealed no significant differences in time to PRL normalisation (males: 13 months [95% CI 6.1–19.9] vs. females: 8 months [95% CI 3.8–12.2]; log‐rank test: *χ* ^2^ = 1.952, *p* = .162) (Figure 2A) or significant tumour shrinkage (males: 53 months [95% CI 33.4–72.6] vs. females: 40 months [95% CI 29.8–50.2]; log‐rank test: *χ* ^2^ = 1.449, *p* = .229) (Figure 2B). When stratified by tumour size, no significant difference was observed in time to PRL normalisation between micro‐ and macro‐tumours (14 months [95% CI 6.1–21.9] vs. 8 months [95% CI 4.6–11.4]; log‐rank test: *χ* ^2^ = 1.417, *p* = .234) (Figure 2C). In contrast, micro‐tumours demonstrated a shorter time to significant tumour shrinkage compared to macro‐tumours (36 months [95% CI 22.9–49.1] vs. 65 months [95% CI 41.2–88.8]; log‐rank test: *χ* ^2^ = 6.146, *p* = .013) (Figure 2D).

Partial CAB resistance (biochemical and/or radiological) occurred in 40% of patients, including combined resistance in 17%, without significant sex differences. In univariate logistic regression analyses, none of the clinical, hormonal, or radiological baseline variables was associated with CAB resistance. Conversely, higher initial CAB dose was associated with lower odds of resistance; each 1‐mg/week increase in initial dose was associated with a reduction in resistance odds (OR 0.11, 95% CI 0.02–0.69, *p* = .018). Higher cumulative CAB dose at 12 months was associated with increased odds of resistance (OR 1.017 per mg, 95% CI 1.001–1.033, *p* = .043).

### 3.3 Treatment discontinuation and recurrence

Overall, 53 (48.2%) of patients experienced CAB withdrawal during the study period, including 5 women due to pregnancy and planned discontinuation following progressive down‐titration after disease remission in 26 cases. Twenty‐two patients discontinued first‐line CAB due to either resistance (*n* = 11), poor compliance (*n* = 5), or intolerance (*n* = 6).

Eleven CAB‐resistant patients underwent surgery after 39 ± 33 months; 8 (5 males, 4 microprolactinomas) had combined resistance, while 3 females (one microprolactinoma) had biochemical resistance. Surgical outcomes are discussed below. Discontinuation due to spontaneous pregnancy occurred after 106 ± 16 months in women with prior tumour shrinkage (complete disappearance in two) and PRL normalisation (except in one case) during first‐line CAB. Following pregnancy, one maintained remission; three had recurrence but achieved remission after CAB reintroduction; one non‐compliant patient required surgery, achieving remission. Of the patients discontinuing CAB due to poor compliance, one interrupted follow‐up and four required CAB reintroduction, achieving disease control in two cases and requiring surgery in two previously resistant cases.

**FIGURE 3:**
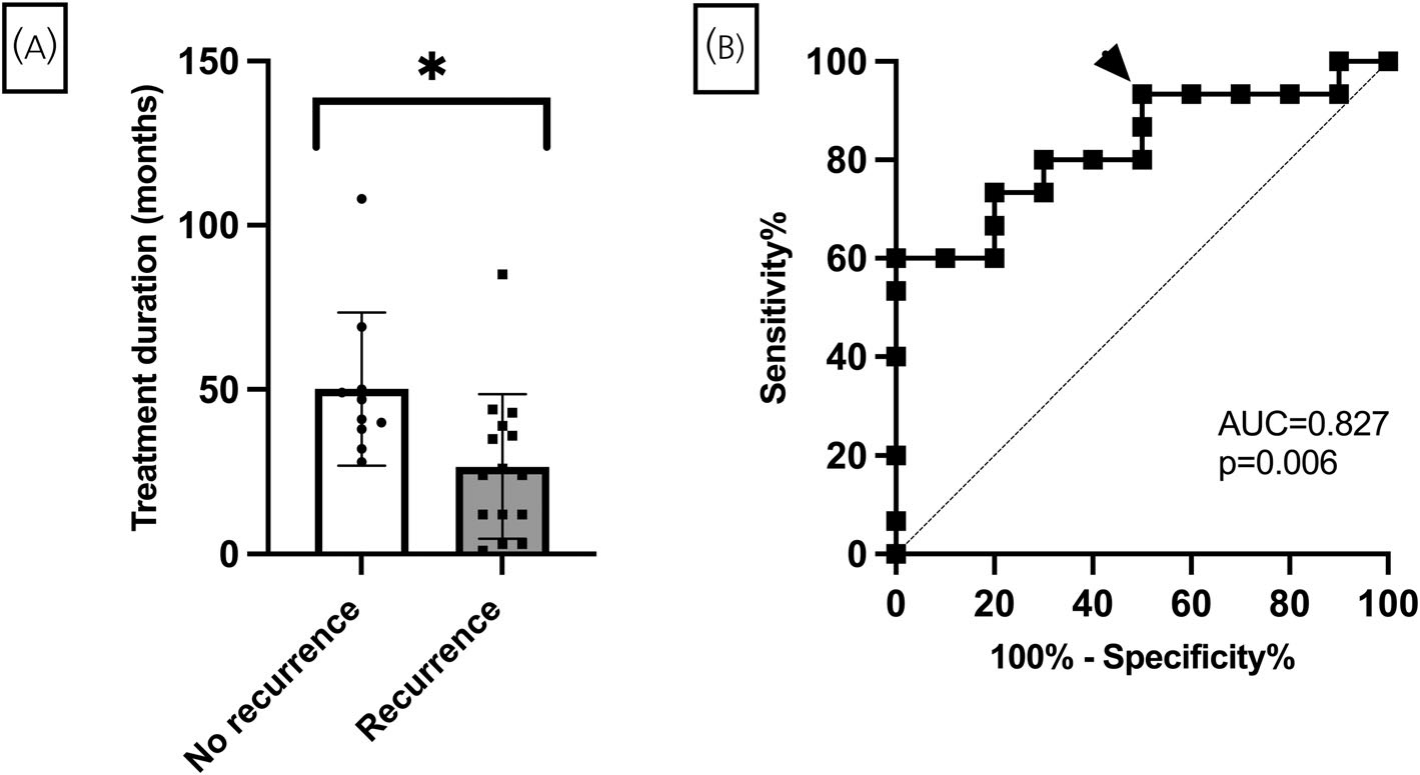
Treatment duration before cabergoline withdrawal and risk of hyperprolactinemia recurrence. Association between treatment duration before cabergoline discontinuation and recurrence of hyperprolactinemia. (A) Treatment duration in patients with and without recurrence after cabergoline withdrawal. Bars represent central tendency with individual patient values shown. (B) Receiver operating characteristic (ROC) curve evaluating the ability of treatment duration to discriminate recurrence after withdrawal, showing good discriminative performance (AUC 0.83; *p* = .006). The green arrow indicates the sensitivity‐oriented treatment duration threshold (45.5 months), corresponding to a sensitivity of 93% and a specificity of 50%.

Among 26 patients undergoing planned withdrawal (mean duration: 36 ± 25 months) after prolonged PRL normalisation (22 with significant shrinkage, 4 complete disappearance), 16 recurred (3 with tumour growth) after a median of 5 months (0.5–52). Recurrent patients were younger (20.1 ± 2.8 vs. 22.7 ± 2.2 years, *p* = .023) and were treated for a shorter duration (26.6 ± 21.9 vs. 50.5 ± 23.2 months, *p* = .017, Figure 3A). In logistic regression, younger age (OR 0.67/year, 95% CI 0.46–0.98, *p* = .040) and shorter treatment duration (OR 0.95/month, 95% CI 0.90–0.99, *p* = .050) predicted recurrence. Treatment duration discriminated recurrence well (AUC 0.826, 95% CI 0.66–0.99, *p* = .006, Figure 3B); a duration shorter than 45.5 months was associated with an increased recurrence risk (sensitivity 93%, specificity 50%). In multivariable analysis, age remained significant (OR 0.54/year, 95% CI 0.30–0.97, *p* = .038) while duration showed a borderline association (OR 0.93/month, 95% CI 0.87–1.00, *p* = .059). All recurrent cases received CAB until the last available follow‐up; two later discontinued successfully.

### 3.4 Safety and adverse events

CAB‐related adverse events occurred in 12% of patients after a median of 6 months [2–7], a cumulative dose of 9 mg [4–14], and a weekly dose of 0.75 ± 0.3 mg, including: dizziness (*n* = 4), nausea/vomiting (*n* = 3), headache (*n* = 2), sleepiness (*n* = 2), hypotension (*n* = 2), irritability/hyperactivity (*n* = 1), tachycardia (*n* = 1), and new‐onset mitral insufficiency (*n* = 1). Six discontinued after 6.5 months (3–39) at 0.75 ± 0.4 mg/week; two underwent surgery (followed by radiotherapy in one case), achieving remission. One refused surgery, used *agnus‐castus*, became pregnant, and achieved remission. Three achieved spontaneous remission. In the remaining seven cases with milder events, all showed spontaneous improvement over time or received a dose reduction. One male patient with a giant prolactinoma (1%) had CAB‐induced apoplexy at 3 months (weekly dose: 0.5 mg, cumulative dose: 6 mg), requiring surgery.

### 3.5 Surgical treatment and pathology

Individual surgical indications and outcomes, along with pathology findings, are detailed in Table S1, Supporting Information. Nineteen patients underwent surgery, five of whom (all males) as first‐line for visual impairment. Immunohistochemistry revealed PRL expression in 16/19 specimens (84%). Silent GH co‐expression was observed in two cases (10.5%); Pit1 immunoreactivity was detected in five cases (26%), including one hormone‐negative specimen. CAB‐resistant and sensitive samples did not differ in Ki‐67 (4% ± 2% vs. 3% ± 2.5%, *p* = .833) or p53 expression (2.5 ± 2.0 vs. 1.0 ± 0.5, *p* = .363), mitotic count (*p* = .581) or PRL immunoreactivity (*p* = .822).

Surgical remission (normoprolactinaemia, no residual tumour) was achieved in 31% of cases. No baseline or treatment‐related variables predicted surgical success. In the patients requiring post‐operative CAB, weekly CAB dose could be significantly decreased in pre‐operatively treated patients (3.5 ± 1.0 vs. 1.0 ± 0.5, *p* = .007), allowing, at the last follow‐up, for remission with no need for treatment in three cases. Moreover, three patients underwent reoperation, two for persistent CAB resistance (later achieving remission), one for intolerance, later requiring radiotherapy leading to tumour stability and normoprolactinaemia. Additional three patients, all of whom were aged ≤18 years at diagnosis, received radiotherapy for uncontrolled disease after surgery; all achieved PRL normalisation with progressive down‐titration of weekly CAB dose. Therefore, radiotherapy was deemed necessary in a minority of patients (4/110 overall, 3.6%), but in 4/19 of operated cases (21.0%).

### 3.6 Long‐term outcomes

At the last available evaluation, median follow‐up was 72 months [15–217], with a median patient age of 27 years [18–36]. CAB therapy was ongoing in 80 patients (72.7%), at a mean weekly dose of 1.5 ± 0.75 mg and median cumulative dose of 144 mg [25–1106]. Of these 80 patients, 69 (86.2%) had achieved PRL normalisation. Median PRL was 18 ng/mL [2–72], and 25% (28/110) of patients showed complete radiological resolution. Median residual tumour diameter was 4.5 mm [0–12]. No case of aggressive or metastatic evolution was observed.

From a clinical point of view, headache persisted in 4/110, and one male patient complained of persistent monocular blindness. In addition, menstrual abnormalities persisted in 6/66 of females, galactorrhoea in 14/47 patients, and sexual dysfunction in 2/18 patients (both males). Spontaneous pregnancy was achieved in 16 female patients (20.8%).

Glucocorticoid replacement was present in 6/110 patients, levothyroxine in 10/110, testosterone replacement in 10/33 of males, and oestrogen replacement in 6/77 of females. Chronic post‐operative desmopressin replacement was required in 10% of operated patients.

Cardiometabolic comorbidities showed minimal change from baseline to last follow‐up. Obesity prevalence remained stable (13% at last follow‐up; *p* = 1.000), as did hypertension (2% [*p* = 1.000]) and dyslipidaemia (1% [*p* = 1.000]). No patient developed type 2 diabetes. Visual deficits persisted in one male patient exhibiting monocular blindness.

## 4 DISCUSSION

This study is the first to systematically characterise prolactinomas diagnosed during the transition age (15–25 years), providing novel insights into clinical presentation, treatment response, resistance patterns, predictors of recurrence after withdrawal, and long‐term outcomes in a large Italian multi‐centre cohort. Unlike previous paediatric series that included younger children or focused exclusively on patients under 18–20 years,^3^, ^4^, ^6^ our cohort captures the critical transition phase from paediatric to adult endocrinology care—a period associated with unique physiological and psychosocial challenges.^8^ This is in line with recent consensus recommendations highlighting the transition age as a distinct clinical phase requiring dedicated management strategies, especially for pituitary disorders.^14^, ^15^

Compared to adult prolactinomas, our cohort exhibited a higher proportion of macro‐tumours and giant tumours, despite a similar rate of tumour invasiveness (lower than 30%^1^). This confirms that transition‐age prolactinomas display a more severe phenotype,^3^, ^4^, ^9^ although less severe than patients under 14 years, where Zhao et al. reported 91.3% macro‐tumours and a 78.3% male predominance.^22^ In this context, our transition‐age cohort represents an intermediate phenotype between paediatric and adult populations, while confirming the established sex differences in clinical presentation. Males presented with significantly larger tumours, higher PRL levels, and greater invasiveness, including all five instances of giant prolactinomas, consistent with previous reports in paediatric and adolescent cohorts.^6^, ^23^ Males also more frequently presented with mass‐related symptoms, while females exhibited hyperprolactinaemia‐related symptoms, consistent with established patterns.^3^, ^4^ Notably, sexual dysfunction was reported in 48% of males but only 2% of females—a finding not systematically reported in prior paediatric series given the younger age of those cohorts.^3^, ^4^, ^5^ This discrepancy likely reflects under‐reporting or under‐investigation of sexual dysfunction in female patients, highlighting the need for comprehensive assessment in both sexes. The high prevalence of baseline amenorrhoea relative to documented gonadotroph deficiency (53% vs. 14% of females) reflects the predominantly functional nature of the hypogonadism: hyperprolactinaemia suppresses hypothalamic GnRH pulsatility causing anovulation, rather than structural gonadotroph insufficiency.^17^ Consistent with a functional mechanism, amenorrhoea was secondary in the great majority of cases and only 6/66 females had persistent menstrual abnormalities at last follow‐up.

CAB achieved PRL normalisation in 75% and significant tumour shrinkage in 66% of patients, arguing for a slightly lower overall efficacy compared to adults, where normalisation occurs in 83%–90%.^1^ According to the current 2023 Pituitary Society Consensus Statement definition,^12^ we found partial resistance in 40% of our cohort, exceeding the 22% reported in the largest paediatric series^3^ and the 10%–20% commonly observed in adults.^1^, ^24^ The higher rate of CAB resistance in our study may reflect the methodological heterogeneity in defining resistance in previous studies, along with a broader definition encompassing either biochemical or radiological resistance; of note, the rate of combined resistance (both biochemical and radiological) in our cohort was 17%, consistent with the 10%–26% reported in paediatric series.^3^, ^9^, ^16^

In our study, higher initial CAB doses were associated with lower resistance odds (OR 0.11); however, this observation is likely biased by higher starting CAB doses in less severe cases and lower starting doses in large/giant macro‐tumours, where rapid shrinkage may be associated with complications such as haemorrhage or cerebrospinal fluid leakage.^25^, ^26^ Similarly, the association between higher cumulative CAB dose at 12 months and resistance probably reflects dose escalation in refractory patients rather than a predictive or causal effect, and should not be interpreted as an actionable predictor. Histopathological markers traditionally associated with aggressiveness and resistance in adult prolactinomas, including Ki‐67 and p53 expression,^1^, ^27^ did not differ between responsive and resistant tumours in our surgical samples. Although limited by sample size, this finding suggests that resistance mechanisms in transition‐age patients may not be fully explained by classical proliferation indices. DA resistance has been linked to alterations in D2 receptor expression^27^, ^28^ and downstream signalling,^29^, ^30^ along with the upregulation of the focal adhesion (FA) signalling pathway.^31^ In addition, reduced oestrogen receptor (ER) expression—particularly ERα—has been associated with a more aggressive phenotype and diminished DA sensitivity, especially in men.^32^, ^33^, ^34^ Given the established role of oestrogens in regulating lactotroph proliferation,^35^ PRL synthesis, and dopamine receptor signalling,^36^ alterations in ER‐mediated pathways may contribute to tumour dedifferentiation and therapeutic resistance. Lastly, a recurrent somatic *SF3B1* R625H hotspot mutation has been identified in a subset of prolactinomas and has been associated with higher PRL secretion and more aggressive tumour behaviour.^37^ Nevertheless, the retrospective clinical design of the present study precludes mechanistic conclusions regarding the high observed resistance rate.

A key novel finding in our study is the identification of treatment duration <45.5 months as a predictor of recurrence after CAB withdrawal, with 93% sensitivity and 50% specificity. Of 26 patients discontinuing CAB after prolonged normalisation, 61% experienced recurrence, consistent with the 50%–68% recurrence rates reported in adult studies^38^, ^39^ and in a recent meta‐analysis of 3564 patients.^40^ In adult studies, full tumour regression with initial PRL <132.7 ng/mL, post‐treatment PRL <1.9 ng/mL,^41^ low maintenance doses,^40^ and the absence of cavernous sinus invasion^39^ have been associated with lower odds of recurrence following discontinuation. Conversely, evidence on DA withdrawal in young patients with prolactinomas remains scarce, with a recent review pooling only 40 patients diagnosed before 18 years across five studies (33% of which reached withdrawal conditions) and a pooled remission rate of 54% after discontinuation,^42^ similar to our cohort, which represents one of the largest transition‐age withdrawal experiences reported to date. In our study, shorter treatment duration and younger age at diagnosis independently predicted recurrence (OR 0.67 per year), supporting the concept that earlier disease onset might be associated with a higher intrinsic risk of relapse, even after sustained biochemical control. As current guidelines recommend attempting withdrawal after a minimum of 2 years in adults,^1^, ^43^ our data suggest that patients with transition‐age prolactinomas may benefit from a longer treatment duration before safe discontinuation. This observation aligns with recent guidelines emphasising the lack of robust evidence for standardised withdrawal criteria in young patients and supporting a cautious, individualised approach.^14^, ^15^

Furthermore, no sex‐based differences in time to PRL normalisation or tumour shrinkage were observed in our transition‐age cohort, consistent with adult data showing comparable biochemical and tumour response between sexes after 3–6 months of CAB therapy.^44^ Regarding tumour size, microprolactinomas achieved tumour shrinkage significantly earlier than macroprolactinomas, while time to PRL normalisation did not differ. Although adult studies report similar *rates* of tumour shrinkage between micro‐ and macroprolactinomas, comparative data on *time* to shrinkage by tumour size are lacking. Whether this finding reflects the greater absolute volume reduction required in larger tumours or distinct tumour biology in transition‐age patients warrants prospective investigation.

Adverse events occurred in 12% of patients at relatively low doses, with 5.5% discontinuing treatment, lower than the 55% reporting moderate side effects in paediatric series, advocating for a “start low, go slowly” approach with careful neuropsychiatric monitoring in young patients,^5^, ^45^ but consistent with adult data,^1^, ^46^ suggesting overall safety in this age group. Notably, one female patient developed mitral insufficiency after 130 months and a remarkably high cumulative dose (969 mg), though cardiac valvopathy is rare at conventional doses ≤2 mg/week.^47^, ^48^, ^49^

Surgery was performed in 17% of patients, with long‐term remission achieved in 31%, closely matching the 33% reported by Yang et al.^3^ and consistent with a recent review reporting 44% remission in macroprolactinomas.^46^ Post‐surgical remission rates were substantially higher in non‐invasive tumours, aligning with prior studies in adolescent cohorts.^3^ Despite post‐operative CAB requirement in over two‐thirds of cases, surgery generally allowed disease control with lower weekly doses, confirming that even in this age group, surgical debulking can restore DA sensitivity.^27^, ^50^ Radiotherapy was reserved for a small subgroup with persistent disease and achieved biochemical control, confirming its role as a third‐line option in selected patients.^51^ Notably, patients requiring multimodal therapy were diagnosed at ≤18 years, further supporting the association between earlier disease onset and a more aggressive clinical course.

At last follow‐up, 73% remained on CAB, of whom 86% had achieved PRL normalisation, with complete radiological resolution in 25% of cases. Cardiometabolic parameters remained stable, with obesity prevalence unchanged, contrasting with recent reports of 39%–49% obesity prevalence in adolescents with untreated hyperprolactinemia^13^, ^52^ and potentially reflecting the beneficial metabolic effects of CAB treatment.^53^ Spontaneous pregnancies were documented in a substantial proportion of females, and permanent hypopituitarism was relatively uncommon, suggesting that timely diagnosis and treatment may mitigate long‐term endocrine sequelae.

The limitations of the study include the retrospective design, potential referral bias from tertiary centres—likely enriching the cohort for larger, invasive and more complex tumours—heterogeneity in treatment protocols and in the evaluation of pituitary function, particularly concerning the somatotroph axis. Moreover, recurrence analyses should be regarded as hypothesis‐generating, as they were based on limited subgroups, potentially reducing statistical power and warranting a prospective validation. Genetic testing was performed in less than 30% of patients, all negative. Given that pathogenetic variants in MEN1 or *AIP* are found in 4%–15% of young patients with macroprolactinomas,^54^, ^55^ incomplete genetic evaluation limits interpretation of resistance patterns and their contribution to recurrence. The strengths of this study include the large sample size, specifically focused on the transition age, long follow‐up duration, and comprehensive assessment of multiple outcomes, including recurrence predictors.

In conclusion, prolactinomas during the transition period demonstrate high CAB resistance rates, frequent need for multimodal therapy, and a substantial recurrence risk after treatment discontinuation, with shorter treatment duration and younger age at diagnosis predicting higher recurrence risk. Sex does not appear to affect biochemical response kinetics, whereas macroprolactinomas require significantly longer to achieve tumour shrinkage. These findings support maintaining CAB as first‐line therapy while recognising the need for individualized treatment duration, careful monitoring for resistance, and readiness to employ surgical and radiotherapeutic options in this challenging population.

## AUTHOR CONTRIBUTIONS

**Dario De Alcubierre:** Conceptualization; investigation; writing – original draft; methodology; writing – review and editing; data curation; formal analysis; software. **Claudia Giavoli:** Writing – review and editing; data curation. **Tiziana Feola:** Writing – review and editing; data curation. **Lorenzo Cerroni:** Writing – review and editing; data curation. **Ludovica Vincenzi:** Data curation; writing – review and editing. **Eriselda Profka:** Writing – review and editing; data curation. **Aurora Pedroli:** Writing – review and editing; data curation. **Giulia Puliani:** Writing – review and editing; data curation. **Rosa Pirchio:** Writing – review and editing. **Marta Tenuta:** Writing – review and editing; supervision. **Renata S. Auriemma:** Writing – review and editing; supervision. **Maria Luisa Appetecchia:** Writing – review and editing; supervision. **Rosario Pivonello:** Writing – review and editing; supervision. **Giovanna Mantovani:** Writing – review and editing; supervision. **Andrea M. Isidori:** Supervision; project administration; resources. **Marie‐Lise Jaffrain‐Rea:** Supervision; writing – review and editing. **Ashley Grossman:** Visualization; validation; project administration; supervision. **Emilia Sbardella:** Funding acquisition; validation; project administration; supervision; visualization; resources.

## CONFLICT OF INTEREST STATEMENT

Andrea M. Isidori reports grants and personal fees from Recordati HRA, Pfizer, and Gedeon. All other authors declare no conflicts of interest.

## ETHICS STATEMENT

The study was approved by the Ethics Committee of Sapienza University of Rome (protocol no. 6525). Written informed consent was obtained from all participants.

## Supporting information


**Table S1.** Pathology data in transition age patients undergoing pituitary surgery for prolactinoma.

## Data Availability

The datasets generated and/or analysed during the current study are available from the corresponding author upon reasonable request.
