# Supplementary material for: Prolactinomas in the transition from adolescence to young adulthood: A multicentre, retrospective study from the TALENT group
Source: J Neuroendocrinol. 2026 Sep 24;38(10):e70275. doi: 10.1111/jne.70275 (PMC13613006; doi:10.1111/jne.70275)
Supplement: Supplementary file 1 [file JNE-38-e70275-s001.docx]

**Supplementary Table 1. Pathology data in transition age patients undergoing pituitary surgery for prolactinoma.**

| **Patient number** | **Sex** | **Age** | **Surgical  timing** | **Indications for surgery** | **Post-operative complications** | **PRL expression** | **GH expression** | **Ki67 (%)** | **P53 (%)** | **Mitoses (HPF)** | **Pathology notes** | **Therapeutic outcomes** |
| --- | --- | --- | --- | --- | --- | --- | --- | --- | --- | --- | --- | --- |
|  |  |  |  |  |  |  |  |  |  |  |  |  |
| #1 | M | 21 | Second-line | Apoplexy during CAB | None | Yes | No | - | - | - | None |  |
| #2 | M | 20 | First-line | Visual impairment | Haemorrhage | Yes | No | 6 | - | 1 | CAM 5.2+ |  |
| #3 | M | 16 | First-line | Visual impairment | None | Yes | No | 6 | - | 0 | Pit1+ |  |
| #4 | F | 17 | Second-line | CAB resistance | None | - | No | - | - | - | Salivary gland * |  |
| #5 | F | 22 | Second-line | CAB intolerance | None | - | No | 6 | 3 | 1 | Pit1+, CK8/18 dot |  |
|  |  |  |  |  |  |  |  |  |  |  |  |  |
| #6 | M | 23 | Second-line | CAB resistance | None | Yes | No | 3 | 0 | 0 | CAM 5.2+ |  |
|  |  |  |  |  |  |  |  |  |  |  |  |  |
| #7 | F | 15 | Second-line | Poor compliance | None | Yes | No | 2 | 1 | 0 | None |  |
| #8 | M | 15 | Second-line | CAB resistance | Hypopituitarism | Yes | Yes | 8 | 4 | 4 | TSH+, Pit1+, CAM5.2+ |  |
| #9 | M | 17 | Second-line | CAB resistance | None | - | No | 1 | 0 | 0 | SYN+, CAM 5.2+ |  |
| #10 | M | 17 | Second-line | CAB resistance | None | Yes | Yes | 2 | 1 | 0 | None |  |
|  |  |  |  |  |  |  |  |  |  |  |  |  |
| #11 | F | 18 | Second-line | CAB resistance | None | Yes | No | 4 | 1 | 1 | None |  |
|  |  |  |  |  |  |  |  |  |  |  |  |  |
| #12 | M | 20 | First-line | Visual impairment | None | Yes | No | - | - | - | None |  |
|  |  |  |  |  |  |  |  |  |  |  |  |  |
| #13 | M | 15 | First-line | Visual impairment | None | Yes | No | 1 | 0 | 0 | None |  |
|  |  |  |  |  |  |  |  |  |  |  |  |  |
| #14 | M | 24 | First-line | Visual impairment | None | Yes | No | - | - | - | Pit1+ |  |
|  |  |  |  |  |  |  |  |  |  |  |  |  |
| #15 | F | 15 | Second-line | CAB resistance | None | Yes | No | 2 | 0 | 0 | CK 8/18+ |  |
|  |  |  |  |  |  |  |  |  |  |  |  |  |
| #16 | F | 20 | Second-line | CAB resistance | None | Yes | No | 3 | **-** | **-** | None |  |
|  |  |  |  |  |  |  |  |  |  |  |  |  |
| #17 | F | 15 | Second-line | CAB resistance | None | Yes | No | - | - | - | None |  |
|  |  |  |  |  |  |  |  |  |  |  |  |  |
| #18 | M | 15 | Second-line | CAB resistance | Hypopituitarism | Yes | No | - | - | - | Pit1+, Cam5.2+ |  |
|  |  |  |  |  |  |  |  |  |  |  |  |  |
| #19 | F | 18 | Second-line | CAB resistance | None | Yes | No | - | - | - | None |  |

Clinical and histopathological characteristics of transition-age patients undergoing pituitary surgery for prolactinoma. Abbreviations: CAB=cabergoline; PRL=prolactin; GH=growth hormone; HPF=high-power field; CAM5.2=cytokeratin CAM5.2; CK8/18=cytokeratin 8/18; Pit1=pituitary-specific transcription factor 1; TSH=thyroid-stimulating hormone; SYN+=synaptophysin immunoreactivity; –=data not available, *details in Feola et al, JEI 2021, doi: 10.1007/s40618-021-01577-6

– =data not available
